# Visible-Light-Driven Photocatalytic Degradation of Dyes and Ciprofloxacin Using Coral-like β-Bi_2_O_3_

**DOI:** 10.3390/molecules31060963

**Published:** 2026-03-13

**Authors:** Thomas Cadenbach, María Isabel Loyola-Plúa, Freddy Quijano Carrasco, Maria J. Benitez, Carlos Reinoso, Alexis Debut, Karla Vizuete

**Affiliations:** 1 Instituto de Energía y Materiales, Departamento de Ingeniería Ambiental, Colegio Politécnico de Ciencias e Ingenierias, Universidad San Francisco de Quito, Quito 170901, Ecuador; marissaloyola@outlook.com; 2 Departamento de Ingeniería Química, Colegio Politécnico de Ciencias e Ingenierias, Universidad San Francisco de Quito, Quito 170901, Ecuador; fquijano@usfq.edu.ec; 3 Departamento de Física, Facultad de Ciencias, Escuela Politécnica Nacional, Ladrón de Guevara E11-253, Quito 170517, Ecuador; 4 School of Physical Sciences and Nanotechnology, Yachay Tech University, Hda. San José s/n y Proyecto Yachay, Urcuqui 100115, Ecuador; creinoso@yachaytech.edu.ec; 5 Centro de Nanociencia y Nanotecnología, Universidad de las Fuerzas Armadas ESPE, Av. Gral. Rumiñahui s/n, Sangolquí P.O. Box 171-5-231B, Ecuador; apdebut@espe.edu.ec (A.D.);

**Keywords:** photocatalysis, emerging pollutants, coral networks, bismuth oxide

## Abstract

Contamination of water bodies caused by increasing human and industrial activities poses a serious threat to human health and environmental sustainability, highlighting the need for green and efficient remediation strategies. In this study, a facile hydrothermal synthesis followed by controlled calcination was developed to fabricate phase-pure α- and β-Bi_2_O_3_ with a unique coral-like hierarchical morphology as visible-light-active photocatalysts. Phase selectivity was achieved by tuning the calcination temperature, yielding pure β-Bi_2_O_3_ while preserving the hierarchical structure. Optical characterization revealed a narrower bandgap for β-Bi_2_O_3_ (2.24 eV) compared to α-Bi_2_O_3_ (2.75 eV), favoring visible-light absorption. Photocatalytic performance was evaluated using Rhodamine B as a model pollutant, where β-Bi_2_O_3_ achieved complete degradation within 240 min, significantly outperforming α-Bi_2_O_3_. The degradation followed pseudo-first-order kinetics, and the catalyst exhibited excellent robustness and reusability. To further demonstrate applicability toward persistent contaminants, Methyl Orange (MO) and the antibiotic ciprofloxacin (CIP) were employed as additional model pollutants. The coral-like β-Bi_2_O_3_ showed high visible-light activity toward MO, including complete removal under acidic conditions. Moreover, efficient degradation of CIP was achieved at neutral pH, with 90% removal within 150 min and complete degradation after 240 min. Overall, these results highlight coral-like β-Bi_2_O_3_ as an efficient standalone photocatalyst for visible-light-driven degradation of dye and pharmaceutical pollutants.

## 1. Introduction

Limited water resources, coupled with inefficient water use and increased demand driven by population growth, pose a significant threat to the world’s finite fresh water supply [1,2]. Anthropomorphic activities such as industrial development, energy production, mining, as well as environmental catastrophes have led to a concerning increase in a large variety of toxic and persistent pollutants in aquatic systems [3,4,5]. Among these, synthetic persistent organic pollutants pose a significant environmental hazard [5,6,7]. In this context, organic dyes, such as Rhodamine B, are of great concern. Organic dyes are commonly used in the textiles, printing, paints, and paper industries. However, their high toxicity, chemical stability, and persistence in aquatic environments define them as a major class of contaminants in industrial wastewater, especially in developing countries [7]. For instance, the textile industry significantly contributes to water pollution, with dye losses ranging from 5% to 50% during different dyeing processes, generating almost 200 billion liters of heavily colored effluents annually [8,9].

The removal of toxic organic substances from aqueous solutions can be addressed by various methods, such as biodegradation, adsorption, and chemical oxidative techniques [10,11,12]. However, the complexity, low efficiency, essential post-treatment, and potential for secondary pollution have proved these methods to be less practical [11]. In recent years, among advanced oxidation processes (AOPs), photocatalytic oxidation of organic pollutants using semiconductors as photocatalysts has gained considerable attention among scientists and engineers [13,14,15]. This growing interest is largely due to the high efficiency in degrading recalcitrant organic pollutants [13,14,15,16]. In photocatalytic processes, the ultimate goal is to achieve complete mineralization of organic contaminants under mild reaction conditions, i.e., converting those pollutants into harmless end products such as CO_2_ and H_2_O [16,17]. In addition, the use of green and sustainable heterogeneous photocatalysis offers distinct advantages over conventional treatment methods. Notably, it requires no additional chemical reagents beyond the semiconductor itself and can operate with energy efficiency, particularly when visible light can be used as the irradiation source [13,14,15,16,17]. Due to these merits, i.e., simplicity, environmental compatibility, cost-effectiveness, mild operating conditions, and the ability to remove multiple pollutants simultaneously, photocatalysis is considered a promising strategy for the degradation of organic contaminants [13,14,15,16,17,18].

Titanium dioxide (TiO_2_) has long been intensively studied as a photocatalyst in environmental applications due to its relatively high photocatalytic activity, excellent chemical and biological stability, low cost, and long operational lifespan [15]. However, its practical application is limited by its wide bandgap, which necessitates the use of energy-intensive and expensive UV light sources. Consequently, increasing attention has been directed toward the development of metal oxide semiconductors that combine non-toxicity and chemical stability with the ability to use visible light for the generation of hydroxyl radicals [19,20].

In this context, bismuth-based photocatalysts such as bismuth oxide (Bi_2_O_3_) have emerged as highly active photocatalysts with bandgaps sufficiently narrow in order to utilize the visible-light spectrum, with applications in catalysis, microelectronics, fuel cells, and gas-sensing technologies [21,22,23]. Bi_2_O_3_ exists in six polymorphic forms, i.e., monoclinic (α-Bi_2_O_3_), tetragonal (β-Bi_2_O_3_), orthorhombic (ε-Bi_2_O_3_), face-centered cubic (δ-Bi_2_O_3_), body-centered cubic (γ-Bi_2_O_3_), and triclinic forms (ω-Bi_2_O_3_) [21,22,23]. Of these, α-Bi_2_O_3_ is the most extensively studied due to its thermal stability and ease of synthesis. However, the application of α-Bi_2_O_3_ in photocatalytic processes is rather limited because of its less negative conduction band (CB) and fast electron–hole recombination rates. Due to these inherent limitations, considerable research has focused on enhancing the photocatalytic activity of α-Bi_2_O_3_ by doping with metal and non-metal elements. However, these modification strategies often involve more complex reaction designs, additional synthesis steps, and increased processing costs, which can limit their practicality for large-scale applications. On the other hand, β-Bi_2_O_3_ has superior photocatalytic performance compared to the a-phase, largely due to its narrower bandgap and improved absorption of visible light. Nevertheless, despite its promising properties, research on the b-phase remains limited, primarily because of the challenges involved in synthesizing this metastable polymorph [22,24,25,26,27,28,29,30].

Furthermore, in the field of photocatalysis, it is well established that not only crystalline phases, i.e., phase purity, but also the size and morphology of semiconductor catalysts have a strong impact on their photocatalytic performance [31,32,33,34]. For instance, smaller crystal sizes generally enhance activity due to increased surface area and more efficient charge transfer at the catalyst surface. It has also been shown often that particle morphology plays a critical role, with specific shapes exhibiting strong correlations with photocatalytic efficiency [32,33]. However, while smaller particles have higher surface area, they do not always guarantee superior activity. For instance, oxygen vacancies and surface defects, which tend to be more pronounced in nanoscale materials, can negatively affect photocatalytic performance by acting as charge recombination centers. Therefore, an optimal balance between size, morphology, and crystalline phase is crucial. Despite the importance of these parameters, facile and reliable synthesis methods that allow precise control over these essential characteristics in the field of bismuth oxide remain scarce.

Herein, we present a facile hydrothermal synthesis method for obtaining phase-pure α- and β-Bi_2_O_3_ with controlled morphology. The synthesized β-Bi_2_O_3_ catalysts exhibited a distinctive coral-like network structure. Remarkably, by simply varying the calcination temperature, we achieved selective phase formation without altering the overall morphology of the material. The synthesized catalysts were applied for the degradation of Rhodamine B (RhB), Methyl Orange (MO), and ciprofloxacin (CIP). These pollutants were specifically chosen to demonstrate the applicability of the coral-like β-Bi_2_O_3_ photocatalyst toward chemically distinct and environmentally relevant contaminant classes, and to enable benchmarking against the visible-light photocatalysis literature. RhB is a widely used cationic xanthene dye probe that allows straightforward monitoring by UV–Vis spectroscopy and is frequently used to compare Bi-based photocatalysts. MO is a representative anionic azo dye with high chemical stability and known persistence, providing a complementary dye structure/charge class to RhB and therefore a more stringent test across dye families. CIP is a fluoroquinolone antibiotic frequently detected in surface waters and wastewater effluents and is associated with growing concerns related to antimicrobial resistance. Compared to dyes, CIP exhibits higher structural stability and more complex degradation pathways, making its photocatalytic removal more challenging. Notably, only a limited number of studies have reported CIP degradation using Bi_2_O_3_-based photocatalysts, underlining both the current interest in this contaminant and the difficulty of achieving efficient degradation under visible-light irradiation.

## 2. Results

**Figure 1:**
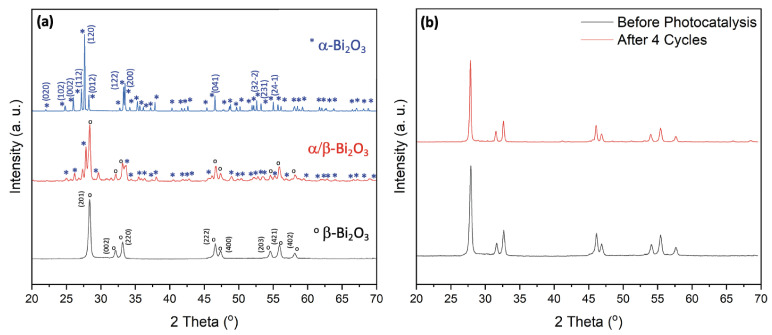
(**a**) XRD patterns for Bi_2_O_3_ demonstrating the influence of calcination temperature on phase formation samples. (**b**) XRD patterns for coral-like β-Bi_2_O_3_ before photocatalysis and after 4 catalytic cycles.

To investigate dependence of the crystalline phase formation on calcination temperature, we calcined the hydrothermally synthesized Bi_2_O_3_ powders (prepared at 120 °C) at four distinct temperatures, i.e., 350 °C, 400 °C, 450 °C, and 500 °C. The corresponding XRD patterns are shown in Figure 1.

The analysis clearly demonstrates that the formation of specific Bi_2_O_3_ phases is strongly influenced by the calcination temperature, which is shown in the direct correlation between thermal treatment conditions and phase development. At 350 °C, the sample exhibits a phase-pure tetragonal β-Bi_2_O_3_ structure, as identified by the characteristic diffraction peaks at 2θ = 28.02°, 31.74°, 32.78°, 46.26°, 47.11°, 54.28°, 55.66°, 57.82°, 74.64°, 75.87°, and 77.82°, corresponding to the (201), (002), (220), (222), (400), (203), (421) and (402) planes, respectively (ICDD PDF# 27-0050). Increasing the calcination temperature to 400 °C does not induce any phase transformation, as the sample remains phase-pure β-Bi_2_O_3_. At 450 °C, however, a phase transition begins to occur. The XRD analysis shows the formation of a mixed-phase system containing both monoclinic α-Bi_2_O_3_ and tetragonal β-Bi_2_O_3_. Finally, at 500 °C, the sample undergoes a complete phase transformation, resulting in phase-pure monoclinic α-Bi_2_O_3_, identified by the major diffraction peaks at 2θ = 25.90°, 26.98°, 27.39°, 33.17°, 35.14°, 37.71°, 46.54°, 52.58°, 58.24°, 61.56°, and 66.86°, corresponding to the (102), (112), (120), (122), (210), (113), (041), (321), (024), (243), and (341) planes, respectively (ICDD PDF# 41-1449). These findings demonstrate that precise phase control in Bi_2_O_3_ can be reliably achieved through careful selection of the calcination temperature, with lower temperatures stabilizing β-Bi_2_O_3_ and higher temperatures promoting the formation of α-Bi_2_O_3_.

**Figure 2:**
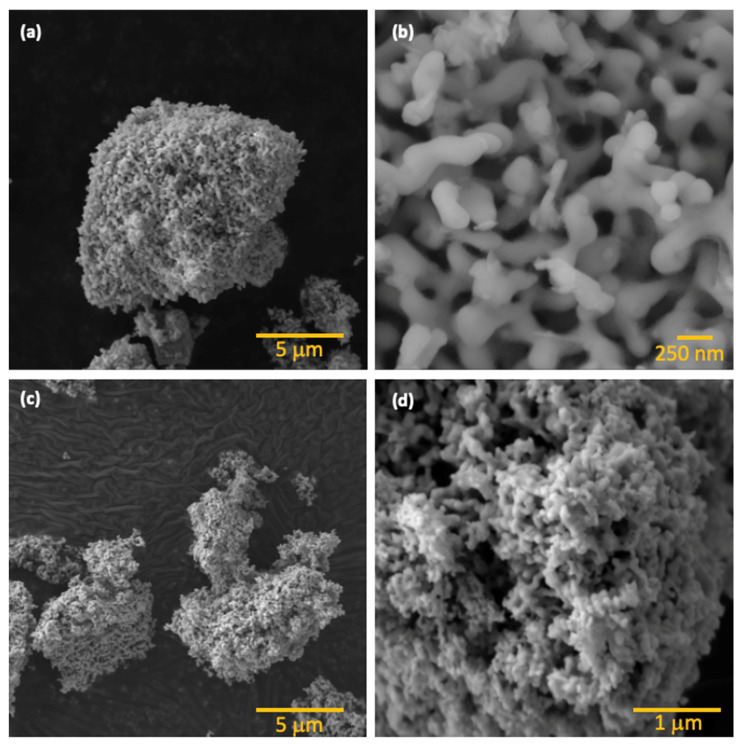
(**a**,**b**) SEM images of coral-like β-Bi_2_O_3_. (**c**) SEM images of α-Bi_2_O_3_ obtained by calcination at 500 °C. (**d**) SEM image of β-Bi_2_O_3_ after 4 photocatalytic cycles.

SEM analysis reveals the formation of branched structures with widths ranging from 100 to 250 nm, as shown in Figure 2. These branches further agglomerate into a highly porous, coral-like hierarchical network, which results in an open framework that could be beneficial for photocatalytic applications by providing a high surface area and enhanced accessibility for reactants. Interestingly, calcination temperature does not significantly influence the morphology of the samples (350 °C, 400 °C, 450 °C, or 500 °C), as all samples retain their characteristic morphology, indicating that the hydrothermal treatment conditions primarily dictate the final structure.

**Figure 3:**
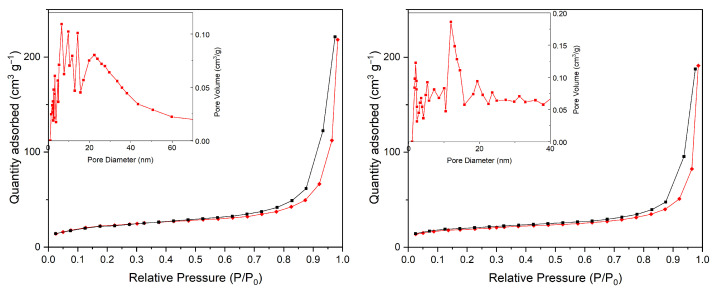
Left: N_2_ adsorption–desorption isotherm and pore size distribution (inset) of β-Bi_2_O_3_ (**left**) and α-Bi_2_O_3_ (**right**).

To further investigate the textural properties of the synthesized Bi_2_O_3_ materials, N_2_ adsorption–desorption isotherm measurements were performed to evaluate the specific surface area, pore volume, and average pore size distribution of selected samples (see Figure 3). The Brunauer–Emmett–Teller (BET) plots are characterized by type IV class isotherms with H4 hysteresis loops indicative of mesoporous materials. The corresponding surface area measurements reveal that the surfaces in the coral-like structure in α-Bi_2_O_3_ and in β-Bi_2_O_3_ are very similar, with 8.7 m^2^/g and 9.9 m^2^/g. The pore size distribution calculated using the BJH method from the adsorption branch reveals a broad mesoporous distribution ranging from approximately 5 to 60 nm. This wide distribution is consistent with the coral-like hierarchical morphology observed in SEM images and suggests that the porosity mainly arises from interparticle voids and structural aggregation rather than uniform templated mesopores.

**Figure 4:**
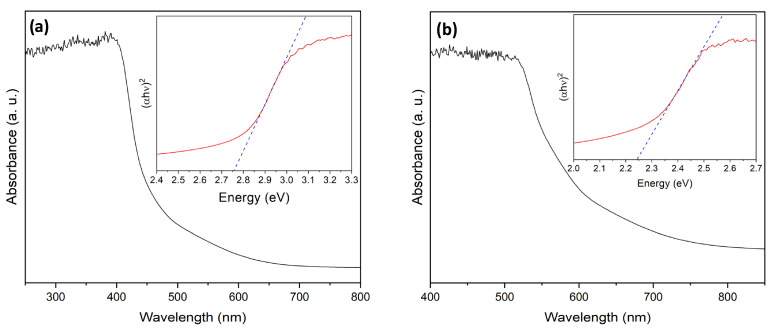
UV-Vis spectra and Kubelka–Munk plots (insets) for α-Bi_2_O_3_ (**a**), and β-Bi_2_O_3_ (**b**).

Since both α- and β-Bi_2_O_3_ polymorphs were successfully obtained in this study, their optical properties were first compared to identify the most promising photocatalyst. The optical properties of the synthesized Bi_2_O_3_ powders were investigated using diffuse reflectance UV-Vis spectroscopy (see Figure 4).

In agreement with previously reported UV-Vis spectra, the UV–Vis spectrum of α-Bi_2_O_3_ displays an absorption band edge at 430 nm, whereas the absorption band edge of β-Bi_2_O_3_ is red-shifted at 540 nm, demonstrating significant visible-light absorption capabilities [21]. The obtained spectra were transformed using the Kubelka–Munk method to determine the bandgap energy. Based on Tauc plots using the equation (αhν)^2^ = A(hν − E_g_), where α is the absorption coefficient, hν is the photon energy, and E_g_ is the bandgap energy, the direct bandgaps of α- and β-Bi_2_O_3_ were determined to be 2.75 eV and 2.24 eV, respectively. The obtained bandgap energies are also in good agreement with previously reported values, which confirms the strong visible and near-UV light absorption capability of Bi_2_O_3_ [21]. It should be noted that bandgap values reported in the literature can vary significantly due to phase purity, oxygen vacancies, and size and shape of particles, which complicates a direct comparison of bandgap values [33,35]. The differences in optical properties between the α- and β-Bi_2_O_3_ polymorphs are also visually evident: α-Bi_2_O_3_ appears white, while β-Bi_2_O_3_ displays a more orange-yellow hue.

In line with previous reports demonstrating the superior visible-light photocatalytic activity of β-Bi_2_O_3_ compared to the α-Bi_2_O_3_ phase, particularly for organic pollutant degradation, β-Bi_2_O_3_ was selected as the primary candidate for detailed surface and electronic structure analysis and subsequent photocatalytic testing. To gain further insight into the surface chemical state and local coordination environment of the active phase, high-resolution X-ray photoelectron spectroscopy (XPS) measurements were performed on the β-Bi_2_O_3_ sample.

**Figure 5:**
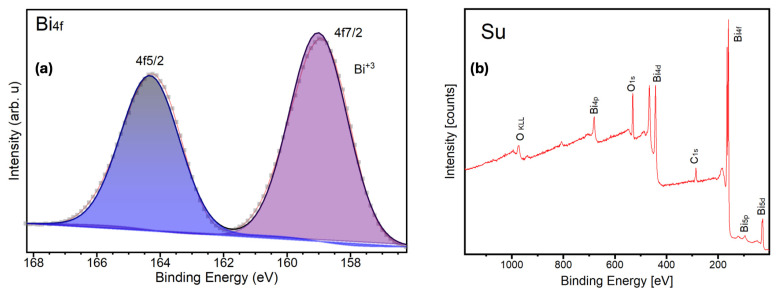
(**a**) Bi 4f core-level XPS spectrum of the sample. The experimental data (dotted line) were fitted using Gaussian components corresponding to the Bi 4f_7_/_2_ and Bi 4f_5_/_2_ spin–orbit doublet. The Bi 4f_7_/_2_ and Bi 4f_5_/_2_ peaks located at 159.2 eV and 164.5 eV, respectively, with a spin–orbit splitting of ~5.3 eV, confirm the presence of Bi^3+^ species characteristic of Bi_2_O_3_. No signals associated with metallic bismuth or reduced bismuth species were detected. (**b**) Survey XPS spectrum showing the elemental composition of the sample.

The high-resolution XPS spectrum of the Bi 4f region exhibits a well-defined spin–orbit doublet with binding energies at 159.2 eV (Bi 4f_7_/_2_) and 164.5 eV (Bi 4f_5_/_2_), corresponding to a spin–orbit splitting of approximately 5.3 eV (see Figure 5a). These values are characteristic of Bi^3+^ species in Bi_2_O_3_ and are in excellent agreement with previously reported Bi 4f binding energies for phase-pure β-Bi_2_O_3_ [36,37]. No features corresponding to metallic Bi^0^ or higher oxidation states were detected, confirming complete surface oxidation of the material. In addition to the binding energy positions, moderate peak broadening observed for both components suggests variations in the local Bi-O coordination environment. Such broadening is commonly associated with structural disorder, oxygen sublattice distortions, or metastable lattice arrangements: effects frequently reported for β-Bi_2_O_3_ and other non-α-Bi_2_O_3_ polymorphs that share the Bi^3+^ oxidation state but differ in crystal symmetry and oxygen coordination [38,39]. Similar effects have been reported for oxygen-deficient or metastable Bi_2_O_3_ phases, where overlapping chemical environments lead to increased line widths rather than distinct chemical shifts [40]. A survey of the XPS spectrum illustrating the overall surface elemental composition of the sample, with prominent Bi core-level photoelectron peaks at characteristic binding energies, confirms the presence of bismuth oxide. The very low intensity of the C 1s signal indicates only trace amounts of adventitious carbon, demonstrating minimal surface contamination and high sample cleanliness (see Figure 5b).

**Figure 6:**
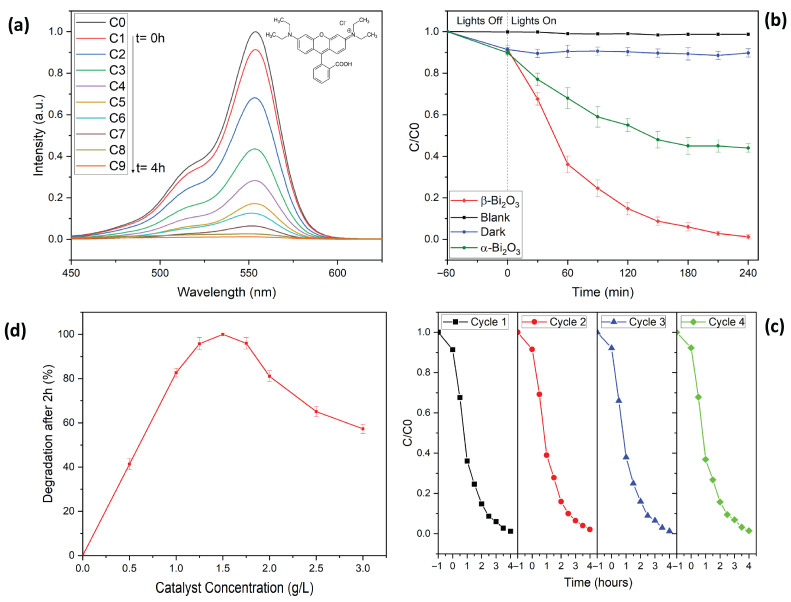
Photocatalytic degradation of Rhodamine B using Bi_2_O_3_ coral networks. (**a**) UV-Vis spectra of Rhodamine B monitored over a total time of 5 h. (**b**) Concentration of Rhodamine B as a function of reaction time. (**c**) Influence of β-Bi_2_O_3_ catalyst concentration on degradation. (**d**) Re-use of the β-Bi_2_O_3_ coral network catalyst in 4 consecutive degradation reactions.

Rhodamine B (RhB), a widely used dye and a persistent organic pollutant, was selected as the model compound to evaluate photocatalytic performance of the phase pure materials, i.e., α-Bi_2_O_3_ and β-Bi_2_O_3_ [7,8,9]. Degradation efficiency was assessed by monitoring the concentration ratio C/C0 over time, where C0 is the initial absorbance, and C is the absorbance at a given irradiation time (see Figure 6).

Notably, RhB remains highly stable under visible light in the absence of a photocatalyst, with no appreciable degradation observed even after 4 h of illumination, as confirmed by the unchanged absorbance peak at 553 nm (Figure 6b) [33].

In order to achieve an adsorption–desorption equilibrium, the reaction mixture was stirred for approximately one hour in the dark. Here, about 8% of RhB was adsorbed onto the catalyst surface. As shown in our previous studies on BiFeO_3_ systems, the dye can be readily desorbed using a solvent mixture of 2-methoxyethanol and water [33,41]. Upon visible-light irradiation (λ = 427 nm and λ = 440 nm), a gradual decline in the RhB absorption peak at 553 nm was observed, indicating effective photocatalytic degradation. After 240 min of visible-light irradiation, the RhB was degraded by 56% when using α-Bi_2_O_3_, while the application of β-Bi_2_O_3_ resulted in the complete degradation of the dye. Additionally, in the case of β-Bi_2_O_3_, no traces of RhB were detected in the post-reaction washing solution, confirming that the dye was fully decomposed rather than simply adsorbed onto the catalyst surface. To the best of our knowledge, this represents one of the highest RhB degradation efficiencies for Bi_2_O_3_-based photocatalysts under neutral pH conditions reported so far [21,22].

This superior photocatalytic performance can be attributed to the combination of phase purity and distinctive structural characteristics of the synthesized materials, which promote more efficient mass transport, improved access to active sites, and enhanced diffusion of dye molecules [21,22]. Additionally, the coral-like network morphology likely contributes to improved light harvesting through internal scattering and reflection effects.

The photodegradation kinetics for β-Bi_2_O_3_ were analyzed using the Langmuir–Hinshelwood (L–H) model, as described by Equation (1).

(1) $$r=-\frac{dc}{dt}=\frac{k_{r}K_{c}}{1+K_{c}}$$

where r is the reaction rate (mg L^−1^ min^−1^), k_r_ is the reaction rate constant (mg L^−1^ min^−1^), K_c_ is the adsorption coefficient (L min^−1^), c is the concentration of RhB (mg L^−1^), and t is the irradiation time (min). For dilute systems, the L–H model simplifies to a pseudo-first-order expression (Equation 2) where C0 is the initial concentration of RhB at time t = 0, C is the concentration at time t, and k is the pseudo-first-order rate constant of photodegradation (min^−1^).

(2) $$\ln\left(\frac{C}{C0}\right)=-kt$$

The obtained rate constant of k = 1.6 × 10^−2^ min^−1^ confirms the excellent photocatalytic performance of the synthesized β-Bi_2_O_3_ coral networks.

In the present study, the pseudo-first-order rate constant for RhB degradation was determined to be k = 1.6 × 10^−2^ min^−1^, which is approximately 2.5 times higher than the value previously reported by our group (k = 6.4 × 10^−3^ min^−1^) [42] (references therein). This improvement demonstrates the positive impact of the modified hydrothermal synthesis and the resulting coral-like morphology on photocatalytic performance.

To further explore optimal reaction conditions, we examined the influence of photocatalyst concentration on RhB degradation by varying β-Bi_2_O_3_ loadings from 0 to 3 g/L (see Figure 6c). At lower β-Bi_2_O_3_ concentrations, the degradation is limited by a lack of sufficient active sites, leading to significantly reduced removal efficiencies. Increasing the catalyst concentration improved performance, with a concentration window of 1.0–2.0 g/L delivering high photocatalytic efficiency. Complete degradation of the organic pollutant was achieved within 2 h at optimal catalyst loading between 1.25 and 1.50 g/L. However, at concentrations above 1.75 g/L, degradation efficiency declined; this can be explained by the increased turbidity of the reaction mixture, which limits light penetration and thus reduces overall removal efficiency [15,33].

We then assessed the recyclability and stability of the β-Bi_2_O_3_ coral networks in four consecutive degradation cycles (see Figure 6d). After each run, the catalyst was recovered by centrifugation and thoroughly washed with methoxyethanol, ethanol and water. After drying overnight, the catalyst was reused under identical reaction conditions using a fresh RhB solution. Remarkably, the photocatalytic efficiency remained nearly unchanged across all cycles, demonstrating excellent stability and recyclability. XRD analysis of the reused catalyst (Figure 1b) confirmed the preservation of its crystalline structure, with no detectable formation of secondary phases and without loss of overall crystallinity. Additionally, the structural robustness of the catalyst is further confirmed by an SEM analysis performed after four consecutive degradation experiments. Here, the overall coral-like morphology remained unchanged (see Figure 2d).

**Figure 7:**
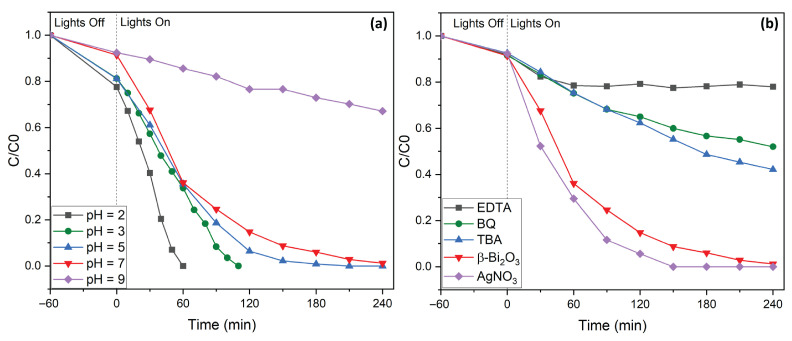
(**a**) Influence of pH on the degradation of RhB by coral-like β-Bi_2_O_3_. (**b**) Trapping experiments in the photodegradation of RhB using coral-like β-Bi_2_O_3_.

To identify optimal conditions for rapid and efficient RhB degradation, photocatalytic experiments were conducted for various levels of pH (pH = 2–9, see Figure 7a). Under basic conditions (pH > 7), degradation efficiency was significantly reduced. This decline can likely be attributed to electrostatic repulsion between the negatively charged hydroxyl groups on the catalyst surface and the anionic carboxylate functionalities of RhB, which hinder effective adsorption and reaction. It has also been reported that under these conditions, RhB dimers are formed due to electrostatic interactions, which influence the charge density throughout the molecule and possibly lead to an interference in the degradation process [43]. Furthermore, hydroxyl radicals, i.e., active species in the degradation process, react at pH > 7 with hydroxy anions. This results in a decrease in the concentration of hydroxyl radicals and the formation of less oxidizing species such as O^−^. In contrast, as the pH decreases toward acidic values, photocatalytic performance improves significantly. At pH levels below the second dissociation constant of RhB (pK_S2_ = 3.22), the dye predominantly exists in its protonated form, which interacts more favorably with the catalyst surface. These conditions promote improved adsorption and, consequently, enhanced degradation kinetics [43,44,45]. Notably, at pH = 2, complete degradation of RhB was achieved within just 60 min (Figure 7a).

**Figure 8:**
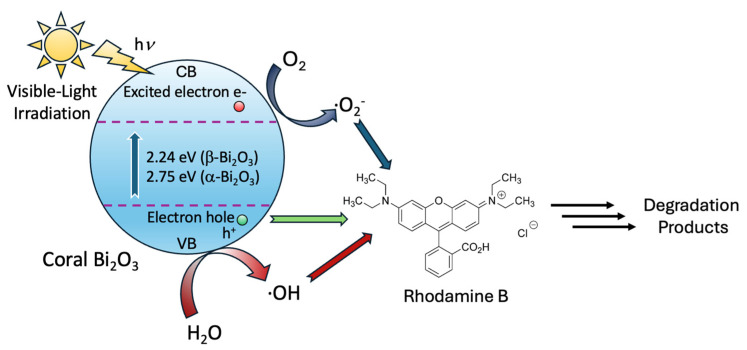
Mechanistic aspects for the photodegradation of RhB using coral β-Bi_2_O_3_.

In order to gain insight into the underlying mechanism of the photocatalytic degradation of RhB by β-Bi_2_O_3_ coral networks, we carried out a series of radical trapping experiments using well-established scavengers (Figure 7b). During the degradation process, a noticeable blue shift in the maximum absorbance peak from 553 nm to 548 nm was observed [38,40]. This shift resulted from the formation of intermediate degradation products, arising from the initial removal of ethyl groups and disruption of the dye’s conjugated chromophore system [22,43,46]. To gain further insight into mechanistic aspects, in particular, the reactive species involved, specific scavengers were introduced into the reaction system. Here, tert-butyl alcohol (TBA, 2 mM) was used to quench hydroxyl radicals (∙OH); benzoquinone (BQ, 0.5 mM), which is a known superoxide radical (∙O_2_^−^), and ethylenediaminetetraacetic acid (EDTA, 2 mM) served as electron hole (h^+^) scavengers. In all cases, the presence of these scavengers led to significant reductions in photocatalytic efficiency, i.e., 42% with TBA, 52% with BQ, and 78% with EDTA. These results indicate that all three species play critical roles in the overall degradation pathway. Furthermore, the addition of silver nitrate (AgNO_3_), which is a known electron scavenger, resulted in an improvement in dye removal efficiency, with complete degradation occurring within approximately 130 min. This can be explained by the trapping of photogenerated electrons by AgNO_3_, which facilitates more effective charge separation and reduces recombination losses. These findings confirm that hydroxyl radicals, superoxide radicals, and photogenerated holes are all actively involved in the degradation process. The observed improvements with electron scavenging further highlight the importance of efficient charge carrier separation. To summarize, upon visible-light irradiation, β-Bi_2_O_3_ absorbs photons, generating electron–hole pairs. Photogenerated electrons in the conduction band reduce dissolved oxygen to form superoxide radicals (•O_2_^−^), while photogenerated holes in the valence band directly oxidize the dye or react with surface-adsorbed water/hydroxide ions to generate hydroxyl radicals (•OH). These reactive oxygen species subsequently attack and degrade the organic pollutant molecules. The improved degradation observed in the presence of AgNO_3_ further confirms that suppressing electron–hole recombination enhances the generation of reactive species (see Figure 8). The overall mechanism is consistent with previously proposed degradation pathways [22,46].

To further probe the photocatalytic performance of the synthesized coral-like β-Bi_2_O_3_ networks, the azo dye Methyl Orange (MO) was selected as an additional model pollutant. Azo dyes represent a major class of environmentally problematic contaminants due to their high chemical stability, toxicity, and potential carcinogenicity, which render them difficult to remove using conventional wastewater treatment technologies [47,48]. MO is extensively used in textile, pharmaceutical, and laboratory applications and is particularly resistant to photodegradation in its deprotonated azo form, which predominates at pH values above its pK_a_ (pK_a_ = 3.4). Under acidic conditions, MO adopts a protonated quinone-like structure that is more reactive and susceptible to oxidative attack. These characteristics make MO a demanding and representative probe molecule for evaluating visible-light photocatalytic activity.

**Figure 9:**
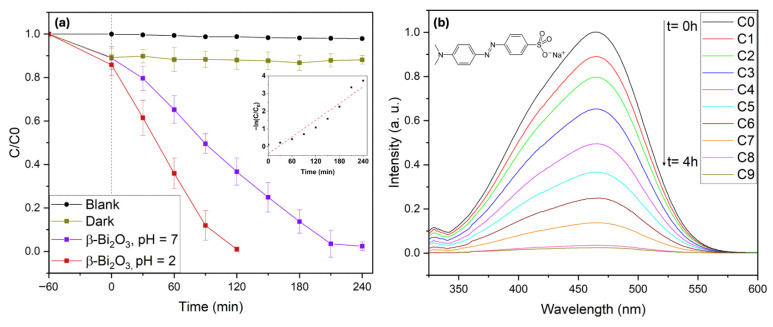
Photocatalytic degradation of Methyl Orange using coral-like β-Bi_2_O_3_ photocatalysts. (**a**) Photodegradation of Methyl Orange as a function of reaction time using coral-like β-Bi_2_O_3_. Inset: Kinetics of photocatalytic degradation of MO using coral-like β-Bi_2_O_3_ at pH = 7. (**b**) UV–Vis spectra of Methyl Orange during the photodegradation.

Photocatalytic degradation experiments were carried out using 10 mg L^−1^ MO solutions under visible-light irradiation (2 × 427 nm and 2 × 440 nm Kessil LED lamps) in the presence of coral-like β-Bi_2_O_3_. In the absence of illumination, approximately 11% of MO was removed during dark adsorption, indicating moderate interaction between the dye molecules and the catalyst surface (see Figure 9). Furthermore, control experiments under visible-light irradiation in the absence of the photocatalyst showed only slight autodegradation of MO (<3%), confirming its high photostability under the applied conditions. Under visible-light irradiation at neutral pH (pH = 7) in the presence of coral β-Bi_2_O_3_, 98% degradation of MO was achieved within 4 h, demonstrating markedly enhanced activity compared to previously reported β-Bi_2_O_3_ systems under comparable conditions [42]. For MO degradation at neutral pH (pH = 7), the coral-like β-Bi_2_O_3_ catalyst exhibited a pseudo-first-order rate constant of k = 1.59 × 10^−2^ min^−1^. This value is higher than many reported standalone β-Bi_2_O_3_ systems, which often require acidic conditions (pH ≈ 2–3) to achieve comparable activity [42,49]. The ability to maintain high degradation kinetics under neutral conditions further highlights the enhanced intrinsic activity of the present catalyst. Notably, under acidic conditions (pH = 2), complete degradation of MO occurred within 2 h, reflecting the increased reactivity of protonated MO and favorable dye–catalyst interactions [42]. These results further underscore the exceptional visible-light-driven photocatalytic efficiency of the β-Bi_2_O_3_ coral networks toward the removal of chemically stable azo dyes, highlighting their strong potential for advanced wastewater remediation applications.

In addition to dye pollutants, the photocatalytic performance of the coral-like β-Bi_2_O_3_ networks was further evaluated using ciprofloxacin (CIP) as a representative pharmaceutical contaminant. Ciprofloxacin is a broad-spectrum fluoroquinolone antibiotic extensively used in human and veterinary medicine for the treatment of bacterial infections. Owing to its widespread consumption and incomplete metabolic degradation, CIP is frequently detected in hospital effluents, municipal wastewater, and surface waters, where it poses serious environmental risks by promoting antibiotic resistance and exerting toxic effects on aquatic organisms. Its chemically stable molecular structure and resistance to biodegradation make CIP a particularly challenging target for conventional wastewater treatment technologies, rendering it a stringent probe compound for advanced photocatalytic systems.

**Figure 10:**
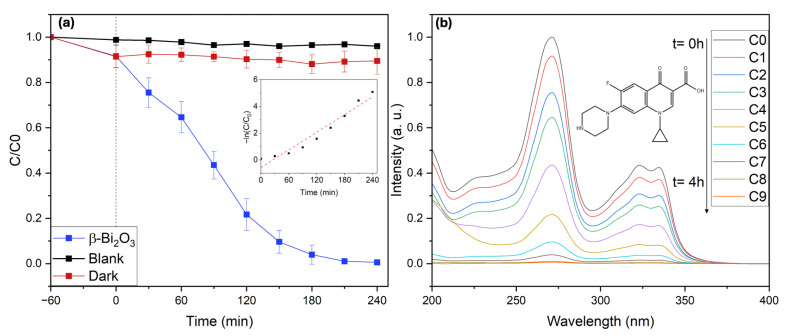
Photocatalytic degradation of CIP using coral-like β-Bi_2_O_3_ photocatalysts. (**a**) Photodegradation of CIP as a function of reaction time using coral-like β-Bi_2_O_3_. Inset: Kinetics of photocatalytic degradation of CIP using coral-like β-Bi_2_O_3._ (**b**) UV spectra of CIP during the photodegradation.

Photocatalytic degradation experiments were carried out using an initial CIP concentration of 10 mg L^−1^ under visible-light irradiation (2 × 427 nm and 2 × 440 nm Kessil LED lamps) in the presence of β-Bi_2_O_3_ coral networks. Control experiments demonstrated that CIP exhibits high photostability under visible-light irradiation in the absence of a photocatalyst, with only ~4% degradation observed over the irradiation period (see Figure 10). It is important to note that the use of ultrapure water is essential when evaluating CIP degradation, as trace organic matter or inorganic ions can promote unintended autodegradation pathways. Dark adsorption tests revealed that approximately 10% of CIP was removed in the absence of light, confirming moderate adsorption of the antibiotic onto the catalyst surface. Under visible-light irradiation at neutral pH (pH = 7), the photocatalytic system achieved 90% degradation within 150 min, followed by complete removal after 240 min, highlighting the high efficiency of the catalyst for antibiotic degradation. For CIP, the coral-like β-Bi_2_O_3_ photocatalyst exhibited a pseudo-first-order rate constant of k = 2.19 × 10^−2^ min^−1^ under visible-light irradiation. Notably, efficient CIP degradation using stand-alone β-Bi_2_O_3_ is only sparsely reported [50,51,52]. In many studies, enhanced antibiotic removal relies on heterojunction or composite engineering. Compared with the pure β-Bi_2_O_3_ baseline rate constant implied in a recent CIP study 8.3 × 10^−3^ min^−1^, our catalyst shows an approximately 2.6-fold higher kinetic constant, highlighting the high intrinsic activity of the coral-like β-Bi_2_O_3_ architecture [52]. The degradation mechanism of ciprofloxacin under visible-light photocatalysis has been comprehensively discussed in the referenced study, and the degradation trends and efficiencies observed here are fully consistent with the proposed reactive-species-driven pathways. The outstanding activity demonstrated by the β-Bi_2_O_3_ coral networks underscores their intrinsic photocatalytic capability, arising from the combination of phase purity, favorable band structure, and highly accessible hierarchical morphology. These findings further highlight β-Bi_2_O_3_ as a highly efficient and rare example of a single-phase visible-light-active photocatalyst for the removal of persistent pharmaceutical pollutants from water.

## 3. Materials and Methods

### 3.1. Characterization Techniques and Equipment

The structure and phase purity of the synthesized materials were characterized using a Bruker D2 Phaser X-ray diffractometer (Bruker, Billerica, MA, USA) with a 1.54184 Å copper tube. Using the DIFRACC.EVA V4.3.1.2 software, a semi-quantitative analysis of the diffraction pattern was performed to identify secondary phases. The morphological analysis of the sample was performed using scanning electron microscopy. A field emission electron microscope, MIRA 3, TESCAN, equipped with a Bruker X-Flash 6–30 detector and a resolution of 123 eV at Mn Kα (Bruker, Billerica, MA, USA), was used. The diffuse reflectance spectrum was measured by UV-Vis spectroscopy (Perkin Elmer, Waltham, MA, USA) with a wavelength range of 200–1000 nm, using an integrating sphere. These spectra were transformed by a Kubelka–Munk model to estimate the bandgap value. The adsorption–desorption isotherms were recorded on a Quantachrome Autosorb IQ 6AG/HOB analyzer (Boynton Beach, FL, USA). The Brunauer–Emmett–Teller (BET) equation was utilized to determine the specific surface areas, while the Barrett–Joyner–Halenda (BJH) algorithm was employed to derive the pore size distribution from the head adsorption branches of the isotherms.

### 3.2. Photocatalytic Experiments

The photocatalytic activity of the synthesized Bi_2_O_3_ samples was evaluated at ambient temperature using RhB, MO, and CIP as model pollutants. RhB degradation experiments were conducted using an initial dye concentration of 5 mg L^−1^ at neutral pH (pH = 7). For MO, an initial concentration of 10 mg L^−1^ at pH = 7 was employed, while CIP degradation was investigated using an initial concentration of 10 ppm, also at pH = 7. In all experiments, 50 mL of the pollutant solution was mixed with 50 mg of the Bi_2_O_3_ photocatalyst.

Prior to irradiation, each suspension was magnetically stirred in the dark for 60 min to establish adsorption–desorption equilibrium between the pollutant molecules and the catalyst surface. Photocatalytic reactions were then carried out under visible-light irradiation using four Kessil LED lamps (2 × PR160, λ = 427 nm and 2 × PR160, λ = 440 nm, all 125mW/cm^2^), positioned 10 cm from the center of the reaction beaker. During irradiation, 5 mL aliquots were withdrawn at regular time intervals (every 30 min) and centrifuged at 1500 rpm for 3 min to separate the catalyst. The pollutant concentration in the extracted solution was determined based on the Lambert–Beer equation by measuring the absorbance at the maximum intensity of the characteristic absorption peak. UV–Vis absorption spectra were recorded using a GENESYS™ 30 UV–Vis spectrophotometer equipped with a tungsten–halogen light source and a silicon photodiode detector. Spectral data were processed and fitted using the Thermo Scientific VISIONlite PC software suite (version 5.0).

### 3.3. Synthesis of Bi_2_O_3_ Coral Networks

**Figure 11:**
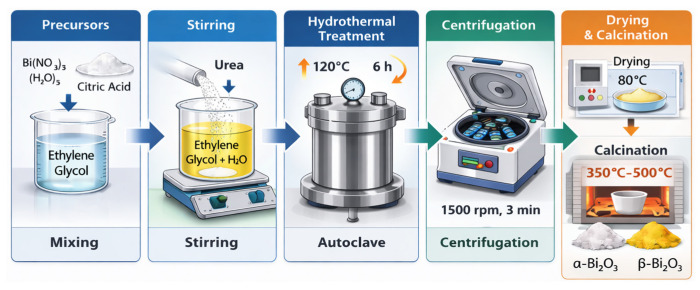
Schematic illustration of the hydrothermal synthesis route for coral-Like α- and β-Bi_2_O_3_.

In a typical synthesis, 12 mmol of bismuth nitrate pentahydrate Bi(NO_3_)_3_(H_2_O)_5_ of purity ≥ 98% (Sigma-Aldrich, St. Louis, MO, USA molecular weight = 485.07 g/mol) and 3 mmol of citric acid of purity ≥ 99.5% (Sigma-Aldrich, molecular weight 192.124 g/mol) were dissolved in 6 mL ethylene glycol C_2_H_6_O_2_ of purity 99.8% (Sigma-Aldrich). After stirring the solution at 500 rpm for 10 min, 0.54 g urea CO(NH_2_)_2_ (molecular weight = 60.06 g/mol) and 114 mL distilled water were added to the reaction mixture. After stirring the solution for 15 min, the sample was transferred into a 300 mL Teflon-lined stainless steel autoclave and heated to 120 °C for 6 h. The autoclave was allowed to cool down naturally to room temperature, and the sample was centrifuged at 1500 rpm for 3 min. After isolating the obtained powder, the sample underwent successive washes with distilled water/ethylene glycol mixtures in a ratio of 6:1, followed by centrifugation. This process was conducted 8 times, with centrifugation increasing by a minute after each washing cycle. Thereafter, the precipitate was collected and dried in a ventilated oven at 80 °C overnight. Ultimately, samples were calcined at 350 °C, 400 °C, 450 °C, and 500 °C with a rate of temperature increase of 1 °C/min and a decrease of 3 °C/min (see Figure 11).

## 4. Conclusions

This work demonstrates a controllable hydrothermal strategy for the synthesis of phase-engineered α- and β-Bi_2_O_3_ with a robust coral-like hierarchical morphology. Precise phase tuning was achieved through calcination temperature control, yielding pure β-Bi_2_O_3_ at 350–400 °C, mixed α/β phases at 450 °C, and pure α-Bi_2_O_3_ at 500 °C without morphological alteration. Structural and surface analyses (XRD, SEM, BET, and XPS) confirmed stable porous networks, while optical characterization revealed a significantly narrower bandgap for β-Bi_2_O_3_ (2.24 eV) compared to α-Bi_2_O_3_ (2.75 eV), enabling enhanced visible-light absorption.

Under visible-light irradiation, coral-like β-Bi_2_O_3_ exhibited markedly superior photocatalytic performance, achieving complete RhB degradation within 240 min, whereas α-Bi_2_O_3_ showed limited activity. The reaction followed pseudo-first-order kinetics, with optimal catalyst loading between 1.25 and 1.50 g L^−1^. The catalyst demonstrated excellent stability over repeated cycles, with an unchanged crystallographic phase after reaction. Mechanistic studies confirmed the involvement of •OH, •O_2_^−^, and photogenerated holes, highlighting efficient charge separation as a key factor governing activity.

Importantly, the catalyst maintained high performance beyond conventional dye pollutants. Efficient degradation of Methyl Orange and antibiotic ciprofloxacin demonstrates applicability toward structurally diverse and environmentally relevant contaminants without reliance on heterojunction architectures.

Overall, this study establishes coral-like β-Bi_2_O_3_ as an effective standalone visible-light photocatalyst, offering phase-controlled synthesis, structural stability, and broad-spectrum pollutant degradation, thereby advancing the development of simple yet high-performance materials for sustainable water treatment.

## Data Availability

The original contributions presented in this study are included in the article. Further inquiries can be directed to the corresponding author.

## Abbreviations

The following abbreviations are used in this manuscript:

RhB	Rhodamine B
MO	Methyl Orange
CIP	Ciprofloxacin
